# Sleeve gastrectomy for acute gastric volvulus: a salvage surgery

**DOI:** 10.1097/RC9.0000000000000848

**Published:** 2026-08-12

**Authors:** Sagar Gyawali

**Affiliations:** Department of General Surgery, Dadeldhura Hospital, Dadeldhura, Nepal

**Keywords:** case report, gastric volvulus, sleeve gastrectomy

## Abstract

**Background:**

Gastric volvulus is a rare but potentially fatal condition in which the stomach rotates along its vertical or horizontal axis. It can present with acute symptoms such as abdominal pain, distension, and retching without emesis, or with chronic, nonspecific symptoms. Volvulus may lead to closed-loop obstruction, ischemia, and perforation. Early diagnosis can be challenging owing to nonspecific symptoms, and delays in treatment may result in gastric necrosis requiring resection.

**Case presentation:**

We present the case of a 64-year-old man with acute, severe epigastric pain and abdominal distension experienced for 1 day. His abdomen was tense and tender on palpation, indicating the need for urgent surgical intervention. Emergency laparotomy revealed organoaxial gastric volvulus with knotting at the gastroesophageal junction and antro-pyloric region, leading to closed-loop obstruction and ischemia of the greater curvature. After derotation, the necrotic segment was resected via a sleeve gastrectomy, and gastropexy was performed.

**Conclusion:**

A high index of suspicion with timely intervention is key to the management of acute gastric volvulus. Management includes derotation of the volvulus and abdominal wall gastropexy in non-ischemic cases or gastrectomy in cases of gastric ischemia.

## Introduction

Gastric volvulus is defined as a rotation of the stomach on itself by at least 180 degrees along its longitudinal or transverse axis. It was first described in 1886 by Berti following an autopsy[1]. The peak age of occurrence of gastric volvulus is the fifth decade, with children younger than 1 year accounting for 10–20% of cases[2]. Organoaxial volvulus occurs when the stomach rotates along the longitudinal axis, and mesenteroaxial volvulus occurs when the stomach rotates along its horizontal axis, with organoaxial volvulus being more common (two-thirds of all cases)[3]. Volvulus is a life-threatening condition, as it can cause closed-loop obstruction, ischemia, and perforation[4]. Here, we present a case of organoaxial gastric volvulus leading to closed-loop obstruction and ischemia that was treated with sleeve gastrectomy. This article has been reported in accordance with the SCARE checklist[5].

## Case history

**Figure 1.**
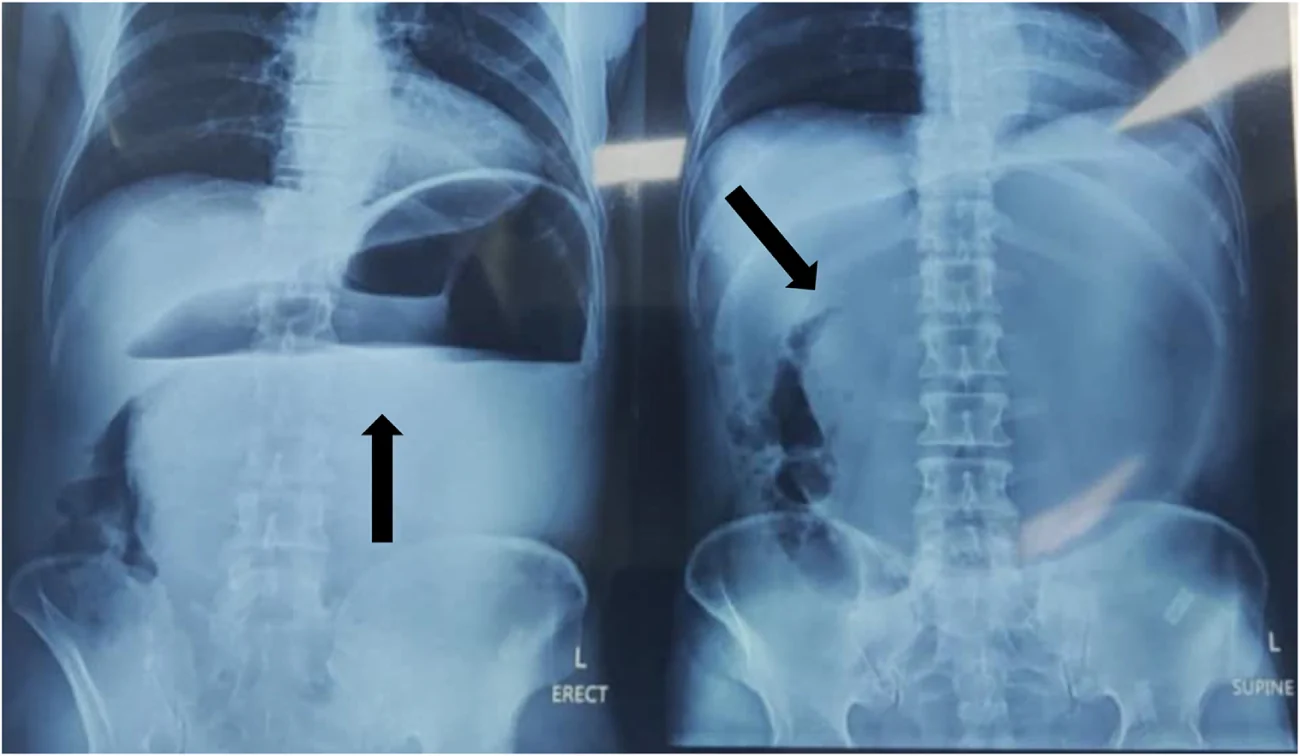
Abdominal X-ray: erect view showing air-fluid levels and supine view showing a bubble-like appearance of the distended stomach.

**Figure 2.**
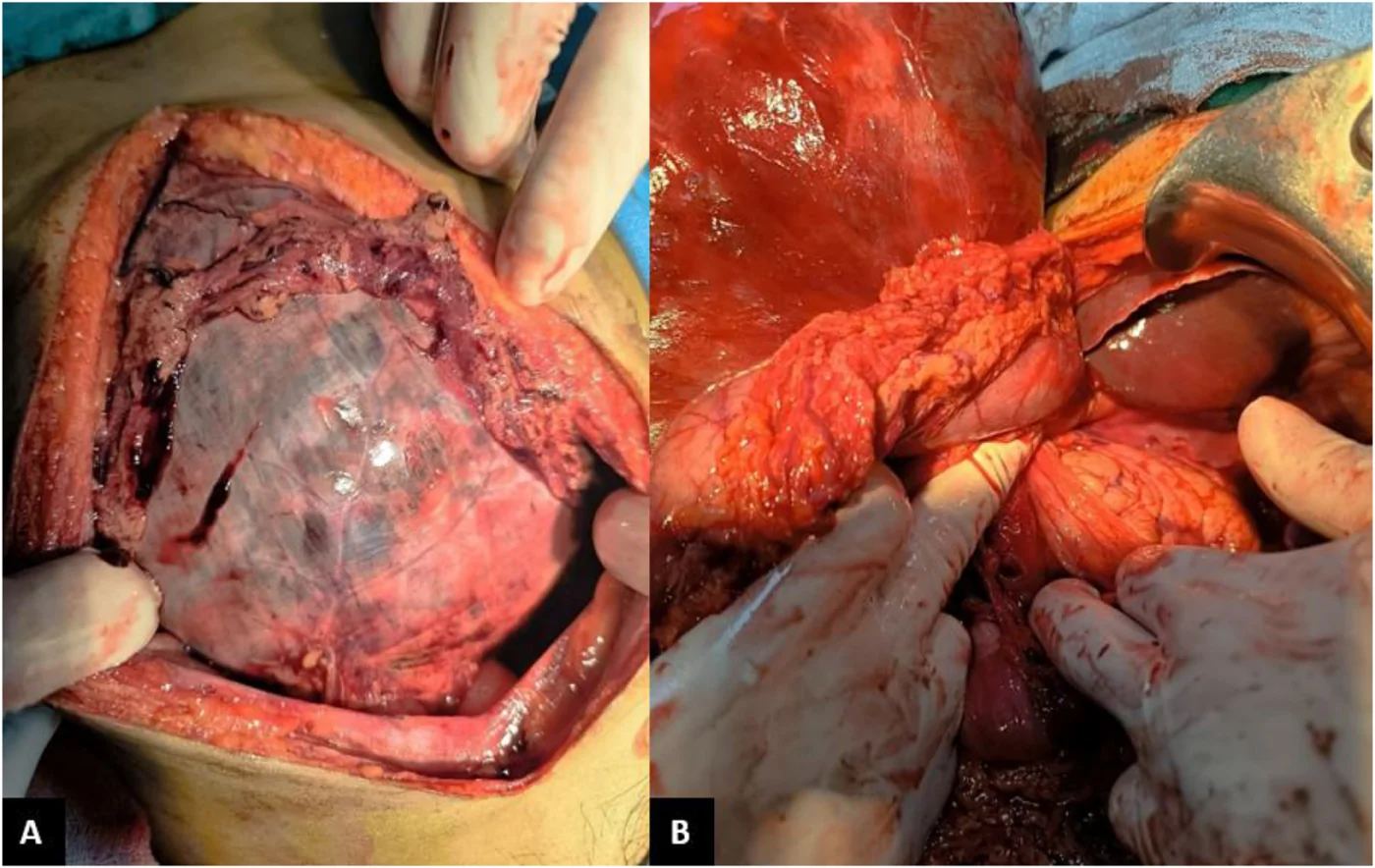
(A) Initial picture at laparotomy showing a distended stomach occupying the whole abdomen and pushing the collapsed bowel loops posteriorly. (B) Knotting at the gastroesophageal junction causing proximal obstruction.

**Figure 3.**
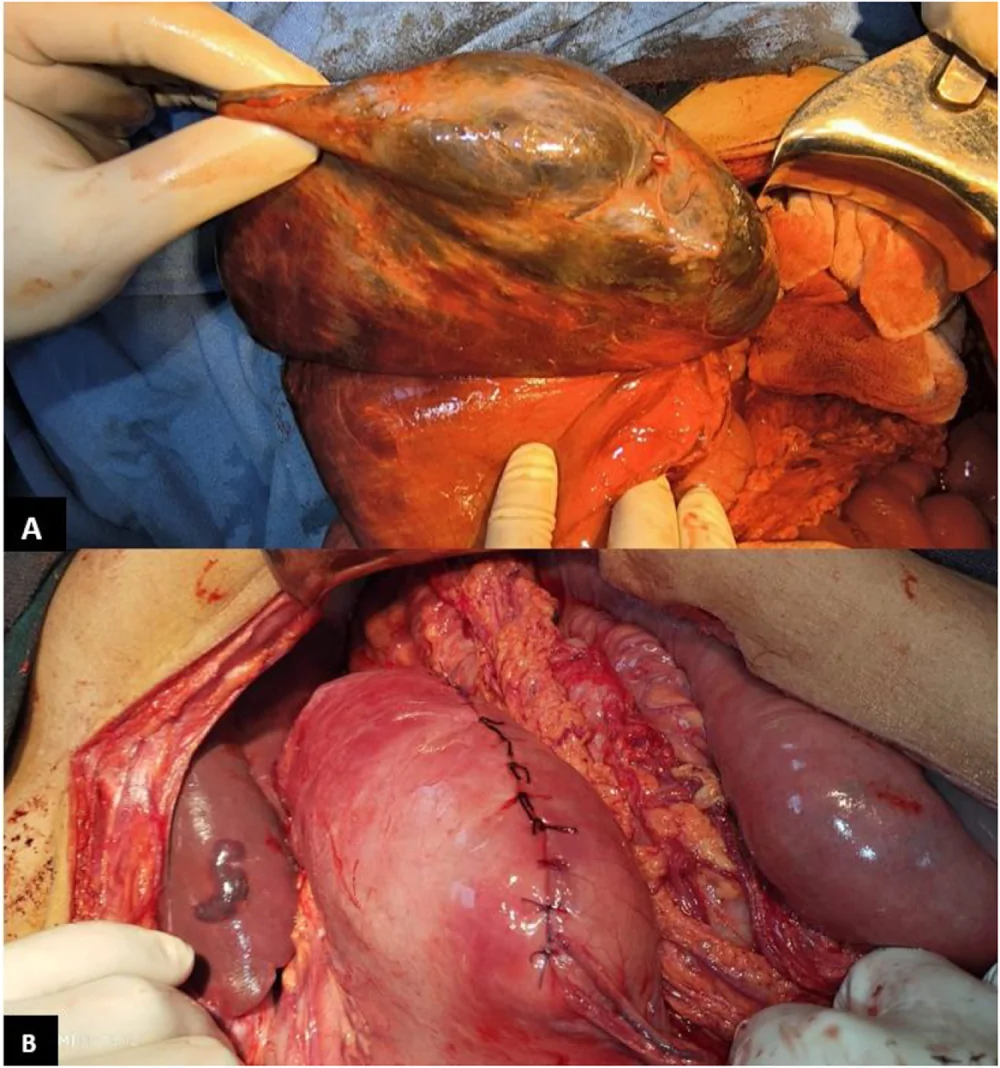
(A) Gangrenous greater curvature of the stomach. (B) Stomach after sleeve gastrectomy to remove the gangrenous greater curvature, with hand-sewn closure of the defect.

A 64-year-old male arrived at the emergency department of our center with complaints of abdominal pain and abdominal distension for 1 day. The pain was colicky in nature, mostly in the epigastric region, and was severe. The patient had passed stool and flatus until the previous day. Pain was associated with abdominal distension. The patient had no comorbidities, no prior surgery and was not under any medications. On examination of the patient’s vital signs, his pulse rate was 105 per minute, blood pressure was 110/70 mm Hg, and he was afebrile. His BMI was 24.2 kg/m^2^. The abdomen was grossly distended on inspection. Upon palpation, the abdomen was tense, tender, and rigid. On digital rectal examination, stool was present in the rectum. Investigations revealed a total leucocyte count of 11 400 with 91% neutrophils, a serum potassium of 2.7 mmol/L, and a blood sugar of 229 mg/dl. Other investigations were within the normal range. Ultrasonography revealed a gaseous abdomen with a poor echogenic window and minimal free fluid in the pelvis. Erect-view X-ray of the abdomen revealed double air fluid levels in the left upper quadrant, and supine X-ray revealed a large single bubble-like appearance occupying most of the abdomen indicating the distended stomach (Fig. 1). Naso-gastric tube insertion was attempted but failed. Considering the clinical finding suggestive of an acute abdomen, exploratory laparotomy was planned rather than further imaging with CECT. The patient was shifted to the operation theatre, and a midline laparotomy was performed. The initial finding was a large cystic lesion occupying the entire abdomen, pushing the viscera to the dorsum of the abdomen (Fig. 2). After extension of the incision, knotting of the gastro-esophageal junction (Fig. 2) and the antro-pyloric region along the longitudinal axis, causing gastric ischemia and marked distension of the stomach, was visualized. Although we could successfully de-rotate the volvulus and release the distension, there was necrosis of the greater curvature of the stomach (Fig. 3). We performed a sleeve gastrectomy of the ischemic segment and gastropexy of the remnant stomach to prevent reformation of volvulus. Greater curvature of the remnant stomach was sutured to anterior abdominal wall with silk suture as a part of gastropexy procedure. Total of three sutures were placed along the greater curvature. Postoperatively, the patient was under the mechanical ventilation and developed metabolic acidosis. The patient expired on first post-operative day due to refractory acidosis.

HIGHLIGHTS

- Acute organoaxial gastric volvulus can rapidly progress to closed-loop obstruction and ischemia.
- Knotting at the gastroesophageal junction and antrum may cause vascular compromise and gastric necrosis.
- Early diagnosis and prompt surgical intervention are crucial to prevent perforation and mortality.

## Discussion

Gastric volvulus is a rare condition arising from the rotation of the stomach beyond 180 degrees on a horizontal or vertical axis. There is no predilection for any gender or race. Volvulus can be primary or secondary based on the etiology. Primary volvulus accounts for about one-third of cases and is associated with abnormalities of the gastric ligaments. Failure of gastric fixation may result from laxity, agenesis, elongation, or disruption of the gastric ligaments. The majority of cases of primary gastric volvulus present with a mesentero-axial configuration and partial bowel obstruction[3]. This is in contrast to our case of primary volvulus with organo-axial rotation and complete obstruction. Secondary causes may involve hiatal hernia, left diaphragmatic eventration, pyloric stenosis, and intra-abdominal adhesions^[^6–9^]^.

The clinical presentation of gastric volvulus varies, ranging from acute symptoms requiring emergency laparotomy to chronic non-specific symptoms causing a diagnostic dilemma. Acute presentation involves severe epigastric or chest pain, retching without emesis, and sometimes hematemesis. The symptoms in chronic volvulus are non-specific and include intermittent gastric outlet obstruction, vague abdominal discomfort, distension, early satiety, gastroesophageal reflux, and dysphagia. In chronic cases, the stomach may volvulize and maintain a partially obstructed state, or intermittently volvulize and spontaneously return to normal configuration, causing a missed diagnosis. Patients may be symptomatic for months to years before presenting for evaluation. The classical Borchardt’s triad is defined as the combination of epigastric pain, inability to vomit, and inability to pass a nasogastric tube. This classical triad is only present in about 70% of patients^[^10,11^]^. Plain radiographs of the abdomen in erect and supine views should be performed in cases with suspected acute gastric volvulus. Contrast-enhanced CT of the abdomen and chest is the diagnostic modality in stable cases. In cases with chronic symptoms, upper GI endoscopy can sometimes aid in detection.

As the stomach has a robust vascular supply, gastric necrosis is a rare presentation of gastric volvulus, as seen in our case. Gastric ischemia is more common in the organo-axial type of volvulus and in patients with a more severe degree of volvulus[3]. In our case, ischemia was evident only in the greater curvature of the stomach. This may be due to huge gastric distension causing elevated intragastric pressure and impaired intramural blood flow, leading to gastric necrosis, as described by Sengoku *et al* in their case report[12].

Once the diagnosis of gastric volvulus is made, initial fluid resuscitation and nasogastric decompression should be attempted. In a few cases, endoscopy may be helpful to assess for ischemia, de-rotate the stomach, place a nasogastric tube, and relieve the volvulus. However, clinicians often fail to insert a nasogastric tube or perform an endoscopy[3]. Management options for gastric volvulus include endoscopic, laparoscopic, endo-laparoscopic, or open techniques.

Different procedures have been performed for ischemia and necrosis depending on the part of the stomach affected. Shukla et al. report performing a total gastrectomy with esophago-jejunostomy for total gastric necrosis and an esophago-pylorostomy with a feeding jejunostomy for partial gastric necrosis[13]. In our case, we observed that the greater curvature of the stomach was necrotic, sparing the lesser curvature and the pyloric region. After derotation and excision of the necrotic segment, the vascularity of the remnant stomach was intact, so we decided to preserve the stomach as in a sleeve gastrectomy. Sutured gastropexy was performed to prevent recurrence of volvulus. Other options to fix the stomach include placement of two percutaneous endoscopic gastrostomy tubes or laparoscopic gastropexy[3].

Complications of gastric volvulus include ulceration, perforation, hemorrhage, pancreatic necrosis, omental avulsion, and splenic rupture with disruption of the splenic vessel[2]. Acute gastric volvulus has a high mortality rate of up to 60% when associated with strangulation[13].

## Conclusion

Acute gastric volvulus is a life-threatening condition leading to a closed-loop obstruction, ischemia, and perforation-peritonitis. Timely suspicion with prompt intervention is necessary for the successful management of this condition. Management of the ischemic segment includes partial gastrectomy with creation of a neostomach or total gastrectomy.

## Data Availability

Not applicable.
